# Scaling and Disagreements: Bias, Noise, and Ambiguity

**DOI:** 10.3389/frai.2022.818451

**Published:** 2022-04-01

**Authors:** Alexandra Uma, Dina Almanea, Massimo Poesio

**Affiliations:** ^1^Computational Linguistics Lab, School of Electronic Engineering and Computer Science, Queen Mary University of London, London, United Kingdom; ^2^Digital Environment Research Institute, Queen Mary University of London, London, United Kingdom; ^3^Turing Institute, London, United Kingdom

**Keywords:** overlapping labels, annotation disagreement, observer disagreement, temperature scaling, model calibration, cost-sensitive loss

## Abstract

Crowdsourced data are often rife with disagreement, either because of genuine item ambiguity, overlapping labels, subjectivity, or annotator error. Hence, a variety of methods have been developed for learning from data containing disagreement. One of the observations emerging from this work is that different methods appear to work best depending on characteristics of the dataset such as the level of noise. In this paper, we investigate the use of an approach developed to estimate noise, temperature scaling, in learning from data containing disagreements. We find that temperature scaling works with data in which the disagreements are the result of label overlap, but not with data in which the disagreements are due to annotator bias, as in, e.g., subjective tasks such as labeling an item as offensive or not. We also find that disagreements due to ambiguity do not fit perfectly either category.

## 1. Introduction

Crowdsourced data are often rife with disagreements between coders. Hence, a variety of methods have been developed for learning from data containing disagreement. In a previous study, the focus was on developing methods for removing items on which annotators disagreed (Beigman-Klebanov and Beigman, 2009), or aggregation methods able to learn “ground truth” from such data (Dawid and Skene, 1979; Smyth et al., 1994; Carpenter, 2008; Whitehill et al., 2009; Hovy et al., 2013) (see, e.g., Sheshadri and Lease, 2013; Paun et al., 2018, 2022; Uma et al., 2021b for review). More recent work however suggests that better results are obtained by methods training directly from data containing disagreements (Raykar et al., 2010; Rodrigues and Pereira, 2017; Peterson et al., 2019; Uma et al., 2020; Fornaciari et al., 2021; Uma et al., 2021b). But another finding emerging from this recent work is that different methods for learning from data containing disagreements work best depending on the dataset (Uma et al., 2021b). One possible explanation for this difference in performance is disagreements can be due to a number of causes, ranging from annotator error to problematic annotation schemes (e.g., with overlapping labels) to genuine item ambiguity to more general item difficulty. An early proposal regarding distinguishing between different types of disagreement was made by Reidsma and Carletta (2008), who showed that disagreements due to **(random) noise**—random annotator errors—affect model training differently from disagreements due to **bias**—annotator-dependent patterns. Such work raises the question of whether it is possible to distinguish between these two types of disagreement (or other types perhaps) so as to decide which method for learning from disagreement is more appropriate for a given dataset.

In early work (Uma et al., 2021b), we considered a number of approaches to identify the type of disagreement that was most typical in a dataset. However, the objective of the measures used in that work is to identify the type of disagreement in a dataset prior to training a model. In this paper, we report on an investigation of the use of an approach inspired by the idea of **temperature scaling** developed by, e.g., Platt (1999) and Guo et al. (2017) to allow a model to *automatically* adapt in the presence of disagreement in the data. We use a range of datasets known to contain disagreements arising from different sources (Uma et al., 2021b) to train models using the state-of-the-art **soft-loss** approach for learning from disagreement (Peterson et al., 2019; Uma et al., 2020, 2021b) and test whether adding automatic temperature scaling improves model performance. We find that the datasets used can be divided into three groups on the basis of the results obtained with the proposed approach. Automatic temperature scaling works well with datasets in which disagreement is mostly due to substantial overlap between the labels such that annotators have to choose a label more or less randomly. By contrast, the approach does not work at all with data in which the disagreements are due to a clear bias, as in, e.g., subjective tasks such as labeling an item as offensive or not, which is known to be affected by the annotators' political views. Finally, with datasets where most or part of the disagreement arises from linguistic ambiguity lie in between these extremes, suggesting that ambiguity may not sit perfectly within a binary distinction such as the distinction between bias and noise proposed by Reidsma and Carletta (2008).

## 2. Methodology: Temperature-Scaled Soft Loss

In this section, we introduce the **temperature-scaled soft loss** approach, which combines the soft loss approach to learning from disagreement we developed in previous work with our own approach to adding temperature scaling in a deep learning model, which we call **automatic temperature scaling**. We first review the soft-loss approach proposed by Peterson et al. (2019) and Uma et al. (2020) and extend soft-loss by including exploration of the suitability of various standard loss functions for soft-loss training. Next, we discuss the (automatic) temperature-scaled soft-loss methodology which involves weighting the soft loss for each item by a learned temperature parameter.

### 2.1. Soft Loss Learning

The soft-loss functions approach to training from data containing disagreement combines using a standard loss function with a probabilistic soft label generated from crowd annotations (Peterson et al., 2019; Uma et al., 2020). To train a model using the soft-loss function approach, a standard loss function such as cross-entropy or squared error is used; but instead of targeting the ground truth viewed as a one-hot label, a **soft label**—a probability distribution over the labels—is generated from the distribution of crowd labels and used as a target for training the machine learning model. We discuss each step in turn.

#### 2.1.1. Generating Probabilistic Soft Labels

While experimenting with a variety of datasets standardly used for learning from disagreement, Uma et al. (2020) showed that for a soft-loss function, the quality of the predictions is dependent on the method used in generating the probabilistic soft labels, which in turn is dependent on the characteristics of the annotation for the dataset. They evaluated two standard label generation functions—the softmax function and the standard normalization function—finding which is best depends on the dataset. Soft labels obtained through standard normalization were found to be preferable for datasets like CIFAR-10H (Peterson et al., 2019), which were annotated by a large number of expert annotations with high observed agreement among them. Soft labels produced using softmax proved instead more suitable for datasets that do not meet these criteria, such as Gimpel et al.'s pos dataset (Plank et al., 2014a) and the labelme dataset (Rodrigues and Pereira, 2017). Uma et al. (2021b) further showed that the best soft label for mixed quality datasets, such as pdis (Poesio et al., 2019), were obtained by using the posterior distribution of a probabilistic aggregation model such as mace (Hovy et al., 2013). For our novel misogyny dataset armis (Almanea and Poesio, 2022), we found that the normalized distribution of the annotators was the best-performing label.

#### 2.1.2. A Suitable Loss Function

Peterson et al. (2019) only used the cross-entropy loss function, hypothesizing that it was uniquely suitable for the task. Uma et al. (2021b) tested a variety of other loss functions, including Kullback-Leibler (henceforth: kl) and (Summed) Squared Error (henceforth: se)^1^. Malinin and Gales (2019) argued that for datasets with high noise due to overlapping labels and resulting in a multi-modal label distribution^2^ reverse KL-divergence is most appropriate if the goal is to maximize prediction accuracy. They tested their hypothesis on synthetic data, comparing reverse KL-divergence as a loss function with (forward) KL divergence, and showed that while KL-divergence is a sensible loss function for datasets with low data uncertainty and target distributions where “correct” labels are available, reverse KL-divergence is more suitable when this is not the case.

Thus, as a preliminary experiment, we tested the hypothesis of Malinin and Gales (2019) with our (non-artificial) data by training soft-loss functions for each task using the best soft label and each of the divergence functions. We additionally tested the other two well-known probability-comparing loss functions—the cross-entropy loss function (ce) already used in Peterson et al. (2019) and Uma et al. (2020, 2021b) and the Squared error function (se) used in Uma et al. (2021b). Soft-loss functions using each of the stated functions can be expressed using the simplified notation:

- Cross-Entropy Soft loss:
  (1) $$\begin{array}{l} CE\left(y_{hum},y_{\theta }\right)=-\overset{n}{\underset{i=1}{\sum }}y_{hum}^{i}\log y_{\theta }^{i} \end{array}$$
- kl Soft loss:
  (2) $$\begin{array}{l} D_{KL}\left(y_{hum}\;||y_{\theta }\right)=-\overset{n}{\underset{i=1}{\sum }}y_{hum}\log\left(\frac{y_{\theta }^{i}}{y_{hum}^{i}}\right) \end{array}$$
- Reverse kl Soft loss^3^:
  (3) $$\begin{array}{l} D_{RKL}\left(y_{\theta }\;||y_{hum}\right)=\overset{n}{\underset{i=1}{\sum }}y_{\theta }^{i}\log\left(\frac{y_{hum}^{i}}{y_{\theta }^{i}}\right) \end{array}$$
- se Soft loss:
  (4) $$\begin{array}{l} MSE\left(y_{hum},y_{\theta }\right)=\overset{n}{\underset{i=1}{\sum }}{\left(y_{hum}^{i}-y_{\theta }^{i}\right)}^{2} \end{array}$$

where $y_{hum}^{i}$ is the target label for an item *i*, the *best soft label*; $y_{\theta }^{i}$ is the model's predicted probability distribution for that item; and *n* is the number of items in the training set.

We experiment with these variations of the soft loss function and note the prediction accuracy of the trained models, especially in reaction to Malinin and Gales's (2019) hypothesis. The best soft loss function is used for experiments in automatic temperature scaling.

### 2.2. Item Weighting Through Automatic Temperature Scaling

One of the most widely adopted approaches to learning from disagreement involves developing methods for identifying **difficult** items–items on which there is an unexpected degree of disagreement among annotators. Such methods typically use statistical inference to infer the difficulty of an item, and then use such difficulty to weigh or filter items classified as intrinsically difficult (refer to, e.g., Carpenter, 2008; Beigman and Beigman Klebanov, 2009; Whitehill et al., 2009) and the discussion of item difficulty approaches in Paun et al. (2022). In the deep learning literature, a number of methods of this type were developed, for which the term **temperature scaling** is often used.

In this paper, we introduce a method of this type, which we called **automatic temperature scaling**, and combine ideas from both temperature scaling and **Platt scaling**. Platt scaling was proposed as a way to calibrate a logistic regression model, i.e., adjust its parameters to reflect uncertainty (Platt, 1999). To calibrate a model, Platt proposes that two **scalar parameters**, a and b ∈ R, be learned by optimizing the negative log-likelihood function over the validation set while keeping the model's parameters fixed. The learned parameters are used to rescale the logits of the model, **z**_*i*_ resulting in outputs, *f*(**x**_*i*_) = σ(*a***z**_*i*_ + *b*).

Temperature scaling is a single parameter variant of Platt scaling (Guo et al., 2017), where a single scalar parameter, *T*, called the **temperature**, is used to rescale logit scores for all the classes, **z**_*i*_, before applying the softmax function. This way, the model's recalibrated probabilities are given as:


(5) $$\begin{array}{l} f\left({\text{x}}_{i}\right)=\sigma \left({\text{z}}_{i}/T\right) \end{array}$$

where σ(·) is the softmax function. When *T* > 1, the entropy of the output probabilities increases, hence “softening the softmax” and evening out the probability distribution. *T* < 1 hardens the softmax, resulting in a peakier (more modal) probability distribution. Finally, *T* = 1 recovers the unscaled probabilities (Guo et al., 2017). The value of *T* is obtained by minimizing the negative log-likelihood on a held-out validation dataset. Because *T* is independent of the class, *j*, and the item, *i*, *temperature scaling does not affect which class is predicted and hence does not affect prediction accuracy*.

**Automatic temperature scaling**, which we propose here, is a natural extension of temperature scaling. It differs from standard temperature scaling in three key ways. First, automatic temperature scaling learns a parameter *vector* *T*_*i*_ jointly as it learns to predict the classes. It does this by learning a network of weights **w**_*T*_*i*__ and biases *b*_*T*_*i*__ such that


(6) $$T_{i}=softplus(W_{T_{i}}x_{i}+b_{T_{i})}$$

This network of weights is disjoint from the network of weights for learning to map inputs to targets. By using Softplus as the squashing the function (as opposed to sigmoid, ReLu, or Tanh) we apply non-linearity to the network without overly limiting the bounds of *T*_*i*_^4^.

The reason for moving from a single scalar parameter to a vectorial parameter, and from a single value for the whole corpus to an item dependent parameter, is that difficulty is very much item dependent—e.g., not all images are equally easy or difficult—and also class dependent: some classes are more easily confused than others, as discussed in more detail in the next section. The vectorial expression of temperature is similar to the one used in **matrix scaling**, an alternative temperature scaling also proposed by Guo et al. (2017)^5^. But unlike in matrix scaling (or Platt scaling, in which more than one parameter is also learned), the parameters are not tuned on a held-out validation set; rather, the model jointly learns classifier and scaling parameters. During training, the model's outputs, $\hat{y_{i}}=f\left(x_{i}\right)$ are computed as follows:


(7) $$\begin{array}{l} f\left({\text{x}}_{i}\right)=\sigma \left({\text{z}}_{i}*T_{i}\right) \end{array}$$

The model's loss is computed using the appropriate soft loss function.

The second key difference is practical in nature but has notable implications. Unlike in temperature scaling, where the logits are divided by temperature *T*, in automatic temperature scaling, the logits are *multiplied* by the temperature; we found this to work better in practice. The consequence is that in automatic temperature scaling, a warmer temperature (higher values of *T*_*i*_) indicates *lower* uncertainty resulting in peakier probabilities, while colder temperatures indicate higher uncertainty resulting in a more even distribution–the opposite to temperature scaling^6^.

The third key difference can be observed from the definition of *T*_*i*_ in Equation (6). Unlike in standard temperature scaling, in automatic temperature scaling, the model does not have a single temperature value; rather, the temperature of any given item is a function of the input vector for the item and the temperature weights of the model, *W*_*T*_*i*__—the logits for each instance are scaled to a different temperature, determined by the model and learned as a function of the input features of the instance. In this way, if the model is able to identify uncertainty for an input item, it will respond by producing a lower temperature value for that item. The converse is also true. Thus, by considering each instance separately, the model is able to produce temperature values depending on how much data uncertainty it perceives for each item.

This third aspect is vital to understanding the anticipated improvement in predictive accuracy using automatic temperature scaling. In datasets with overlapping labels, because the modal class for affected items is arbitrary, models (much like annotators) are likely to disagree with the modal class of the target labels, predicting a different (and possibly equally plausible) modal class for perceived noisy inputs. The temperature lowering for such items results in a flatter predicted probability distribution and has the added effect of decreasing the loss contribution of that item to the overall loss. Consequently, the model penalizes itself less for such items and reduces the loss contribution of the item to the total loss. In this way, automatic temperature scaling can be comparable to cost-sensitive loss (Plank et al., 2014a).

## 3. The Experiments

In this section, we present our experimental design and discuss the datasets and models used for the experiments conducted in this study.

### 3.1. Experiment Design

We conducted the experiments in two phases. First, we experimentally compared the suitability of various standard loss functions for soft loss training as outlined in Section 2.1.2 on several tasks. Then, we extended the best-performing loss function into an automatic temperature-scaled soft loss. For both experiments, we evaluated the models using two evaluation metrics, one hard and one soft.

#### 3.1.1. Hard Evaluation

As a hard evaluation metric, we used accuracy, as done by Peterson et al. (2019) and Uma et al. (2020). We calculated the accuracy of each model's prediction with respect to a standard: the majority vote aggregate of the expert annotators for armis ^7^ and gold labels for the other datasets.

#### 3.1.2. Soft Evaluation

As noted in previous work (Dumitrache et al., 2018; Peterson et al., 2019; Uma et al., 2020; Basile et al., 2021; Uma et al., 2021b), as the realization that gold labels are an idealization growth, so does the awareness that hard evaluation is not sufficient to compare machine learning models on tasks in which disagreements are extensive, and extremely questionable for tasks in which the labels are subjective and therefore it does not make sense a “gold label” exists that the disagreements can be reconciled to. A particularly obvious illustration of this last point is the **misogyny detection** task, related to hate speech detection. In this task, the labels assigned by annotators are very much dependent on their background, i.e., text found misogynistic by a female annotator or a more liberal annotator may not be found misogynistic by a male annotator or an annotator from a more conservative background.

When evaluating tasks containing disagreements, or in which disagreements may be intrinsic, it would seem insightful not to evaluate models against a questionable gold label only, but also against **soft labels** in the sense discussed above (probability distributions over the labels derived from crowd annotations) in which disagreements are preserved. Consequently, in this paper, our models are also evaluated using a soft evaluation metric, cross-entropy. Like Peterson et al. and Uma et al., we compute the cross-entropy between the probability distribution produced by each model and the **best soft label** produced from the crowd distribution (The label that is most appropriate for that dataset, as discussed above). This form of evaluation provides insight into how well the models are able to capture possible disagreements in labeling resulting from the crowd.

### 3.2. Data

We used in this study four disagreement-preserving datasets that have been previously used in research into learning to classify from disagreement (Jamison and Gurevych, 2015; Plank et al., 2014a,b; Uma et al., 2020, 2021a; Fornaciari et al., 2021) and that exemplify different sources of disagreement (An in-depth analysis of the disagreements in these datasets has been carried out by Uma et al., 2021b). In addition, we used an entirely new dataset, armis (Almanea and Poesio, 2022), illustrating a different type of disagreement not considered by Uma et al. (2021b): disagreement due to subjectivity.

#### 3.2.1. The Gimpel et al. POS Corpus

The first example of a corpus containing disagreements due to ambiguity (Plank et al., 2014b) is Gimpel et al.'s (2011) pos dataset (henceforth, pos), which has been often used in research into developing disagreement-aware nlp models (Plank et al., 2014a; Jamison and Gurevych, 2015; Fornaciari et al., 2021; Uma et al., 2021b). The dataset consists of 14k Twitter posts annotated with ground truth pos tags collected by Gimpel et al. (2011) from expert annotators and crowdsourced tags collected by Plank et al. (2014b)—at least five crowdsourced labels per token from 177 annotators.

The workers annotating this corpus often disagree with the ground truth label; the observed agreement (*A*_*o*_, Artstein and Poesio, 2008) for the dataset is 0.73, as computed using the multi-annotator version of Fleiss Kappa (Fleiss et al., 2004).

A typical example of the disagreements found in this corpus is shown below (the token to be tagged is in bold):

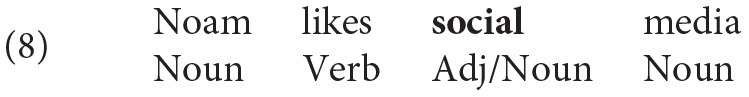



in the context, the category *Noun* would seem to be just as appropriate as the category *Adj* for the token ***social***.

Plank et al. (2014b) conducted an analysis of the easy and hard cases in this dataset, finding that the vast majority of inter-annotator disagreements are due to **genuine linguistic ambiguity**, as in this example, although the pos categories Adj and Noun are clearly distinct, in some cases, it is not possible to tell what is the “right” category (Plank et al., 2014b). In fact, an analysis of the pos dataset carried out by Uma et al. (2021b) showed that the average observed agreement on an “easy” category such as nouns (particularly for name tokens like Twitter handles) is much higher than for other categories.

For experiments using this dataset, we split the 14k tokens into training (12k) and testing (2k) and use the development dataset released by Plank et al. (2014a) for validation.

#### 3.2.2. The PDIS Corpus

The second corpus we used contains disagreements in part due to ambiguity, in part to annotator carelessness. The *Phrase Detectives* 2 corpus (Poesio et al., 2019) is a crowdsourced anaphoric reference corpus collected with the *Phrase Detectives* game-with-a-purpose (Poesio et al., 2013)^8^. Anaphoric reference is another aspect of linguistic interpretation in which ambiguity is rife (Poesio et al., 2006; Versley, 2008; Recasens et al., 2011). For example, Poesio et al. (2006) discussed examples such as (3.2.2).

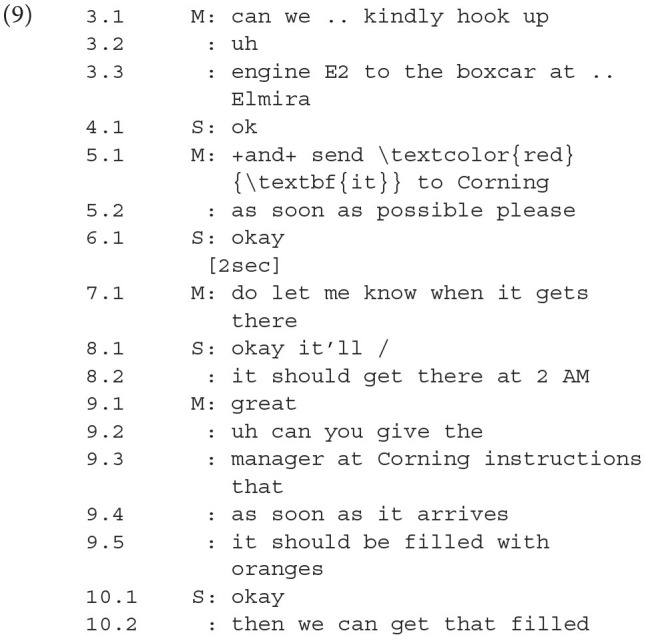



In this exchange, it is not clear whether the pronoun *it* in 5.1 (in red) refers to *the engine E2* that has been hooked up to *the boxcar at Elmira* or to the boxcar itself or indeed whether the distinction matters at all. It is only at utterance 9.5 that we get evidence that *it* probably refers to *the boxcar at Elmira* since only boxcars can be filled with oranges. The two interpretations are clearly distinct–the pronoun cannot refer to both–but it is not possible to decide which is the intended one from the context.

The *Phrase Detectives* 2 corpus consists of 542 documents, for a total of 408K tokens and 107K markables, annotated by slightly less than 2,000 players producing a total of 2.2M judgments—about 20 judgments per markable on average. In total, 64.3% of the markables received more than one distinct interpretation from the players. Some of the disagreements are due to annotator error/carelessness, others to interface issues; but for about 10% of markables, disagreement is again due to **genuine linguistic ambiguity**.

In this study, we used pdis, a simplified version of the corpus containing only binary information status labels: discourse new (DN) (the entity referred to has never been mentioned before) and discourse old (DO) (it has been mentioned). pdis still consists of 542 documents, for a total of 408K tokens and over 96K markables; an average of 11.87 annotations per markable are preserved^9^.

Forty-five of the documents (5.2K markables), collectively called pdgold, additionally contain expert-adjudicated gold labels. This subset of pdis was designated as the test set. The training and development datasets consist of 473 documents (and 86.9K markables) and 24 documents (4.2K markables), respectively^10^.

#### 3.2.3. The LabelMe Corpus

The most widely used corpus for learning to classify images from crowds is the LabelMe dataset^11^ (Russell et al., 2008). It classifies outdoor images according to 8 categories: *highway, inside city, tall building, street, forest, coast, mountain*, or *open country*. Using Amazon Mechanical Turk, Rodrigues and Pereira (2017) collected an average of 2.5 annotations per image from 59 annotators for 10K images in this dataset.

**Figure 1:**
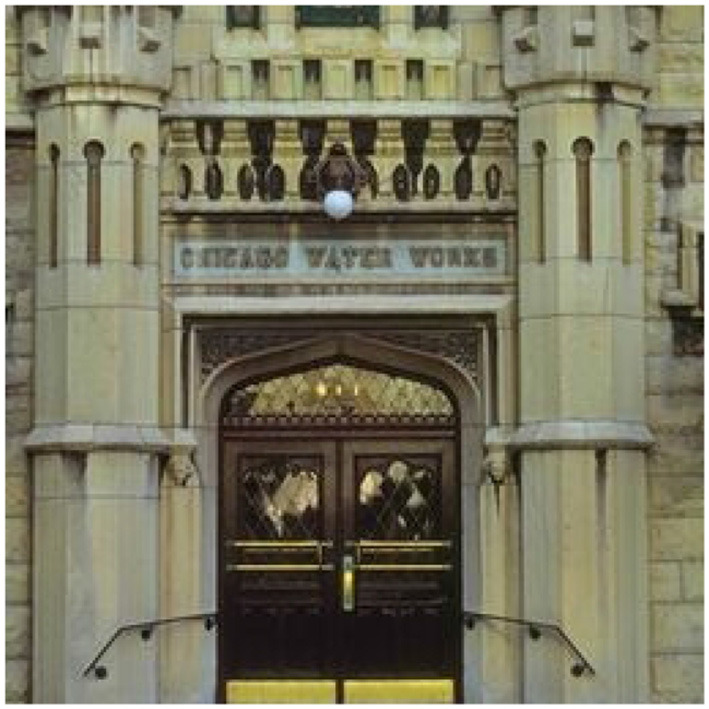
An example of disagreement from LabelMe Ground truth label: *insidecity*, crowd annotations: [*insidecity*:1, *tallbuilding*:2].

The observed agreement for this dataset, also computed using the multi-annotator version of Fleiss et al.'s (2004) Kappa, is 0.73, which is the same level of average observed agreement seen in the pos dataset. However, it can be argued that the source and nature of the disagreement in this dataset are different, consider Figure 1 for an illustration. The ground truth label for the example image is *inside city*, and one annotator chose that label as well, but two other annotators chose *tall building*. Notice the difference from the ambiguity cases in pos and pdis: there, two interpretations are possible, but a word can only have one—it is just that it is not possible to know which from the context. Here, *both* labels can be applied at the same time. Uma et al. (2021b) carried out an analysis of this dataset, finding that examples like Figure 1 are prevalent. That is, the disagreement for this dataset is largely due to an **imprecise annotation scheme** where label categories are not necessarily mutually exclusive but may **overlap**. As a consequence, an annotator forced to choose one among the overlapping categories which apply to a particular image will likely make a random choice.

In our experiments, we randomly split the 10K images into training and test data (8,882 and 1,118 images respectively) to allow for ground truth and probabilistic evaluation. A total of 500 images from the dataset with gold labels were used as a development set.

#### 3.2.4. The CIFAR-10H Corpus

As an example of a crowdsourced corpus containing very little disagreement and that primarily due to item difficulty, we used Krizhevsky's (2009) CIFAR-10H dataset, which consists of 60K tiny images from the web, carefully labeled, and expert-adjudicated to produce a single gold label for each image in one of 10 clearly distinct categories: *airplane, automobile, bird, cat, deer, dog, frog, horse, ship*, and *truck*. Peterson et al. (2019) collected crowd annotations for 10K images from this dataset (the designated test portion) using Amazon Mechanical Turk, creating the cifar-10h dataset^12^, which we use for our experiments.

**Figure 2:**
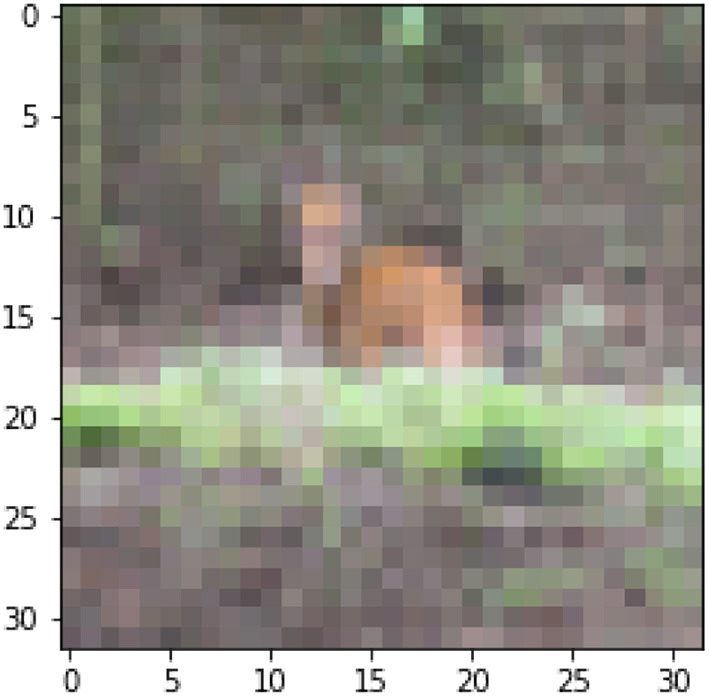
An example of disagreement from cifar10h Ground truth label: *deer*, crowd annotations: [*dog*:33, *deer*:13, *horse*:4].

The observed agreement for this dataset is 0.92, the highest among all the datasets. Clearly, the 2,457 annotators (about 51 annotators per item) found the annotation scheme to be clear and mostly agree with the expert opinion on what the label for each item would be. Notice that unlike in labelme, there is no overlap: it is not possible for an object to belong to multiple categories. Cases of disagreement among annotators do occur, but they are primarily of the kind illustrated by Figure 2, which is because of the poor quality of the image, it is not possible to decide from the picture which animal is illustrated. Yet, there is no question that only one category can apply. We consider such cases as proper examples of **difficult to classify** items—items to which only one category from the scheme applies, yet problematic to classify because of noise.

We used the cifar-10h dataset for training and testing using a 70:30 random split, ensuring that the number of images per class remained balanced as in the original dataset. We also use a subset of Krizhevsky (2009) cifar-10 training dataset (3k images) as our development set.

#### 3.2.5. The ArMIS Corpus

Finally, to exemplify an important source of disagreement—the fact that certain judgments are intrinsically subjective—we used our own armis corpus (Almanea and Poesio, 2022). armis is an Arabic misogyny dataset. It consists of 1K tweets each annotated with binary labels: 1 if the tweet expresses a misogynistic behavior according to the annotator's subjective point of view, 0 if the annotator believes that the tweet is not misogynistic. The tweets were collected using the Twitter API in October 2020, using a keywords list which was manually created specifically for this task, including specific slang words, phrases, and hashtags in order to get the related tweets, such as “Feminist,” “Deficient mind and religion.” The important aspect of this dataset is that it was annotated by three experts” annotators, carefully chosen to reflect different political views: liberal, moderate, and conservative. The annotators were asked to annotate the tweets based on their perspective.

The observed agreement of the annotators is 0.77, higher than the observed agreement of both pos and labelme datasets (0.73), lower than the 0.92 observed agreement of CIFAR-10H, and equal to the observed agreement of pdis. It is important to note while pdis and armis have the same level of disagreement, the nature and source of the disagreement for the armis dataset differs from that of pdis and indeed from the others. While Uma et al. (2021b) show that pdis disagreements can be attributed to noise from spammers, the ambiguity of labels, or interface problems, an analysis of the disagreement in armis showed the nature of the disagreements to be largely due to the **subjective viewpoints** of the diverse annotators.

For these experiments, we split the 964 tweets in armis into 674 for training, 145 for validation, and 145 for testing. Gold labels were not obtained, as is fitting for a task of such as divisive nature, where annotator background plays a substantial role in how they label. However, as a compromise, we use majority voting to produce a hard label for hard evaluation purposes.

### 3.3. Base Models

The base models used in these experiments are the state-of-the-art or near state-of-art models used in previous work (Uma et al., 2020; Almanea and Poesio, 2022), many of which were made available to the participants to the 2021 semeval shared task on learning from disagreement (Uma et al., 2021a). We briefly summarize these models in this subsection.

#### 3.3.1. The POS Tagging Model

For pos tagging, we used the bi-lstm model (Plank et al., 2016) used by Uma et al. (2020). The model we used is improved from Plank et al. (2016) by using attention over the input token and character embeddings to learn contextualized token representations.

#### 3.3.2. The PDIS Information Status Model

The model for this task was also developed by Uma et al. (2021a). Uma et al. combined the mention representation component of Lee et al.'s (2018) coreference resolution system with the mention sorting and non-syntactic feature extraction components of the is classification model proposed by Hou (2016)^13^ to create a novel is classification model that outperforms (Hou, 2016) on the pdis corpus. The training parameters were set following Lee et al. (2018).

#### 3.3.3. The LabelMe Image Classification Model

For the LabelMe image classification, we replicated the model from Rodrigues and Pereira (2017). The images were encoded using pre-trained cnn layers of the vgg-16 deep neural network (Simonyan et al., 2013) and passed to a feed-forward neural network layer with a relu activated hidden layer with 128 units. A 0.2 dropout is applied to this learned representation which is then passed through a final layer with softmax activation to produce the model's predictions.

#### 3.3.4. The CIFAR-10H-10 Image Classification Model

The trained model provided for this task is the ResNet-34A model (He et al., 2016), one of the best performing systems for the cifar-10 image classification. The publicly available Pytorch implementation of this ResNet model was used^14^.

#### 3.3.5. The ArMIS Arabic Misogyny Classification Model

For this task and dataset, we fine-tuned the state-of-the-art AraBERT base model (Antoun et al., 2020) with a maximum sequence length of 128, learning rate of 1e-5, batch size of 8, and training for 10 epochs.

## 4. Results

Table 1 compares the effectiveness of different probability-comparing loss functions for making gold predictions, identifying the best soft loss function for each dataset. Table 2 presents the results obtained for each task by models using the best soft loss function from Table 1 with and without automatic temperature scaling, evaluated using both hard and soft metrics.

**Table 1:** The effect of different loss functions for soft loss training on accuracy.

	**POS**	**PDIS**	**LABELME**	**CIFAR-10H**	**ArMIS**
se Soft loss	79.20	92.90	84.21	63.49	76.83
ce Soft loss	79.80	92.86	84.66	66.54	**77.79**
kl Soft loss	**79.96**	92.86	84.73	**66.58**	76.41
Reverse KL Soft loss	79.81	**92.95**	**84.92**	63.71	75.59

*The bold values indicate the best results for each model (indicated in the first column) using a given metric (indicated in the column header)*.

**Table 2:** Results showing the accuracy (higher is better) and cross-entropy (lower is better) of soft loss models with and without temperature.

**Task**	**Model**	**Accuracy↑**	**Cross-entropy ↓**
LABELME	Reverse KL soft loss	84.97	1.671
LABELME	Reverse KL soft loss + *Ti*	**86.29** ^*^	**1.656**
POS	KL soft loss	79.96	**1.268** ^*^
POS	KL soft loss + *Ti*	**80.01**	1.547
PDIS	Reverse kl soft loss	92.95	0.467
PDIS	Reverse kl soft loss + *Ti*	**93.00**	**0.395** ^*^
CIFAR-10H	KL soft loss	**66.58** ^*^	**1.109** ^*^
CIFAR-10H	KL soft loss + *Ti*	63.89	1.223
ArMIS	CE soft loss	**77.79**	**0.586** ^*^
ArMIS	CE soft loss + *Ti*	76.83	0.636

*An asterisk is used to indicate significantly better results*.

*The bold values indicate the best results for each model (indicated in the first column) using a given metric (indicated in the column header)*.

To account for non-deterministic model training effects, each model was trained and tested several times: (i) 30 times each for pos and labelme (ii) 10 times each for pdis, CIFAR-10H, and armis owing to the complexity of the base models. We measure significance *via* bootstrap sampling, following Berg-Kirkpatrick et al. (2012) and Søgaard et al. (2014). The rest of this section discusses the results from these tables, highlighting significant results. The best result for each dataset is highlighted in bold.

### 4.1. Choosing the Loss Function

The aim of this preliminary experiment was to investigate Malinin and Gales's (2019) hypothesis that Reverse kl divergence is the most appropriate loss function for training models on datasets with high data uncertainty. We found that the Reverse kl soft loss function outperforms the other soft loss functions by a noticeable margin (0.19) for one dataset only, labelme—though this margin is not significant^15^. This is the dataset for which we observe the most disagreement due to an annotation scheme with overlapping labels, as opposed to linguistic ambiguity (as in pos), or a combination of linguistic ambiguity and random noise (as in pdis), or item difficulty (as in CIFAR-10H), or annotator biases (as in armis). For CIFAR-10H, the dataset with the least amount of disagreement (and noise), as discussed by Uma et al. (2021b), we observe that Reverse kl soft loss falls nearly 3 significance points below either ce or kl soft loss. The se loss function also performs poorly on this dataset, likely because se optimizes the loss for non-modal classes, and this is an undesirable trait for a dataset like CIFAR-10H where the modal class is usually the gold class.

Following this experiment, we determine the best soft loss function for each dataset to be used as the starting point for the automatic temperature-scaled soft loss is as follows: ce for armis, kl for pos and CIFAR-10H, and reverse kl for pdis and labelme.

### 4.2. Temperature Scaling Soft-Loss Learning

The first observation emerging from Table 2 is that automatic temperature scaling only significantly improves results in one task: labelme. In other words, our results would suggest that automatic temperature scaling only works when a disagreement arises from overlapping labels, resulting in the arbitrariness of ground truth.

In the next two datasets, pos and pdis, the effect of temperature scaling on the performance of the models are mediocre or non-existent. These are the datasets for which we and Plank et al. (2014b) and Poesio et al. (2019) have shown that although a certain amount of noise is present, the disagreements are largely due to linguistic ambiguity and/or interface limitations.

At the other extreme, we have two datasets in which temperature scaling hurts performance. One of these is CIFAR-10H. This is a dataset with a very high observed agreement, 0.92. We also showed that the very few disagreements in this dataset are due to difficulty experienced by annotators when labeling blurry images. In other words, these disagreements are not systematic or a result of an imprecise annotation scheme but are due to the characteristics of the input. The other dataset for which automatic temperature scaling leads to a reduction in model performance is armis. In this case, there is lower agreement than in CIFAR-10H, but this is not a reflection of systematic noise or data uncertainty, but of annotator uncertainty due to subjective biases.

## 5. Interpreting *T*_*i*_

Our results show that among the datasets we considered in this study, automatic temperature scaling is effective for the one dataset in which disagreements are primarily due to what we may call **label arbitrariness**: the randomness in judgments originating from the fact that annotators have to choose one between multiple labels all of which could apply to an image and do so without appealing to any theory (given the vagueness of the annotation scheme). In this section, we examine the temperature predictions of the model for this dataset to understand what the model learns about label arbitrariness.

**Figure 3:**
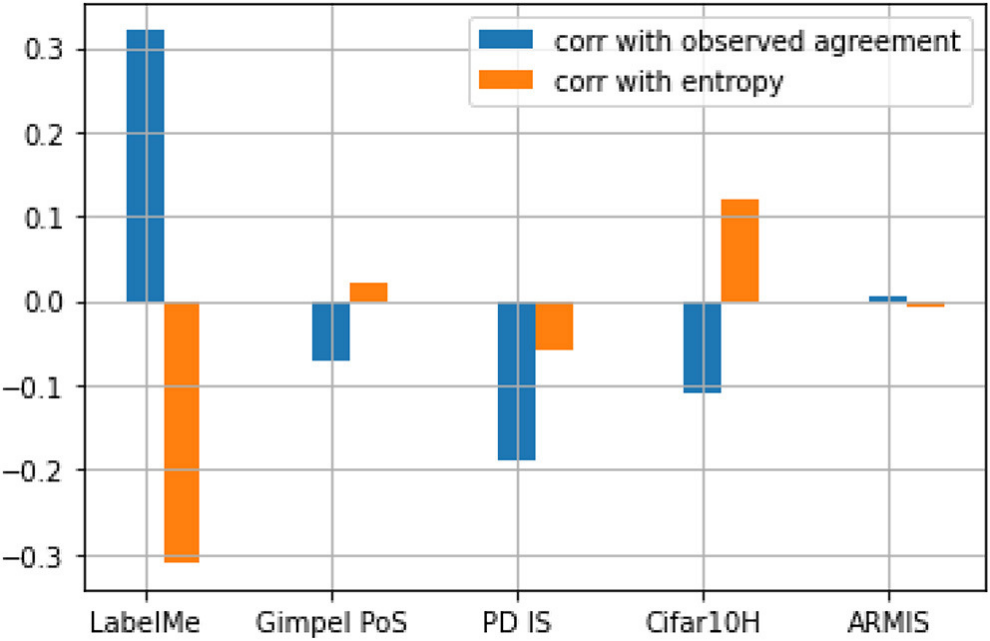
Graph showing the correlation of *Ti* with observed agreement and entropy.

One way to do this is to measure the correlation of the temperature values to known measures of item agreement/uncertainty/difficulty. Figure 3 shows the Pearson correlation (Pearson, 1896) between the temperature parameter and two such metrics of uncertainty/difficulty: observed agreement and normalized entropy. The results show that for labelme, the only dataset for which our method produces a significant improvement over the soft-loss baseline, the model's *T*_*i*_ predictions have the strongest positive correlation to the observed agreement. This means that the model tended to make higher *T*_*i*_ predictions for items with a high observed agreement and lower *T*_*i*_ predictions for items with a low observed agreement. The model also has the strongest negative correlation to entropy. These two results suggest that for this dataset (but not for others), *T*_*i*_ is a moderately good predictor of uncertainty for this dataset as measured by observed agreement and entropy. What is it about the type of disagreement due to annotation schemes in which labels overlap that explains why temperature scaling improves performance with this kind of dataset, but not with others?

As mentioned earlier, in the one study of the differences between types of disagreement we are aware of, Reidsma and Carletta (2008) proposed a distinction between two types of disagreement between annotators and argued that they affect the performance of machine learning models in different ways. One kind is disagreements due to **random noise**, not conforming to any theorizable pattern. A second type is disagreements due to **bias**, which are identifiable through the occurrence of patterns of disagreement. The fact that automatic temperature scaling works best for disagreement due to overlap, which is the type of disagreement among those we studied that most resemble random noise because the annotators have to choose randomly; and it works worst for the clearest case of bias among our datasets, the misogyny data might suggest that automatic temperature scaling is a good method for adjusting model weights when the disagreements are due to random noise, but not when disagreement is due to bias. The mediocre results with pdis and pos suggest that disagreements due to linguistic ambiguity sit somewhere in the middle, or do not fit this distinction at all. Of course, more research is needed to verify if this hypothesis also holds with other datasets in which disagreement is due to noise.

**Figure 4:**
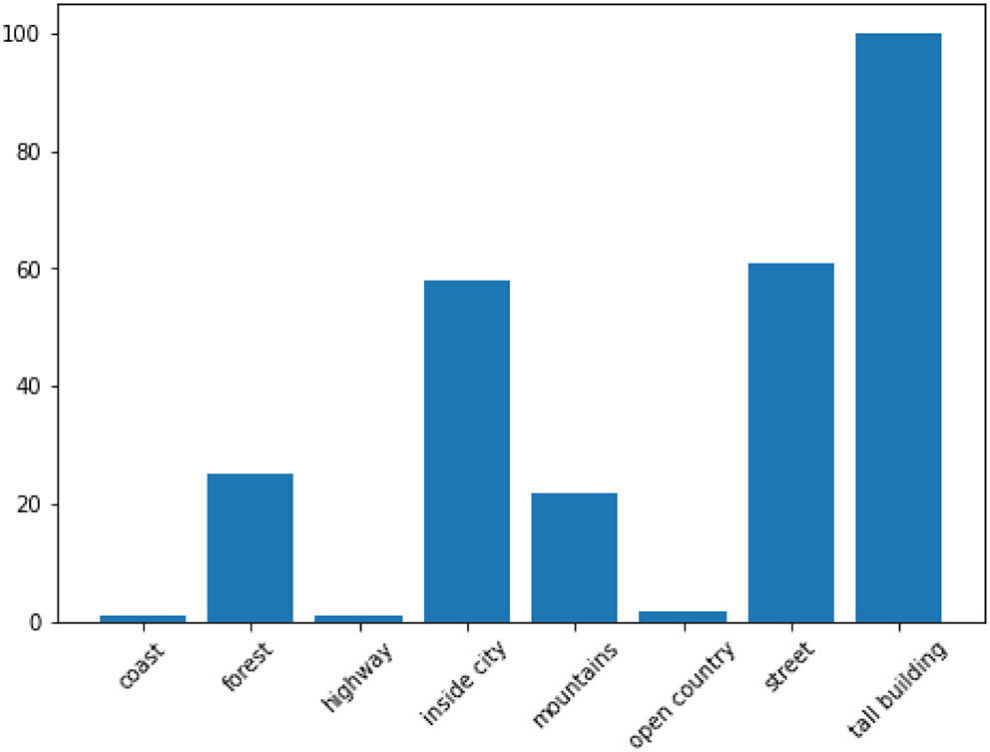
Bar chart showing the gold label distribution of the images with the lowest temperature (images in the 1st quartile range of temperature), i.e., the lowest certainty.

**Figure 5:**
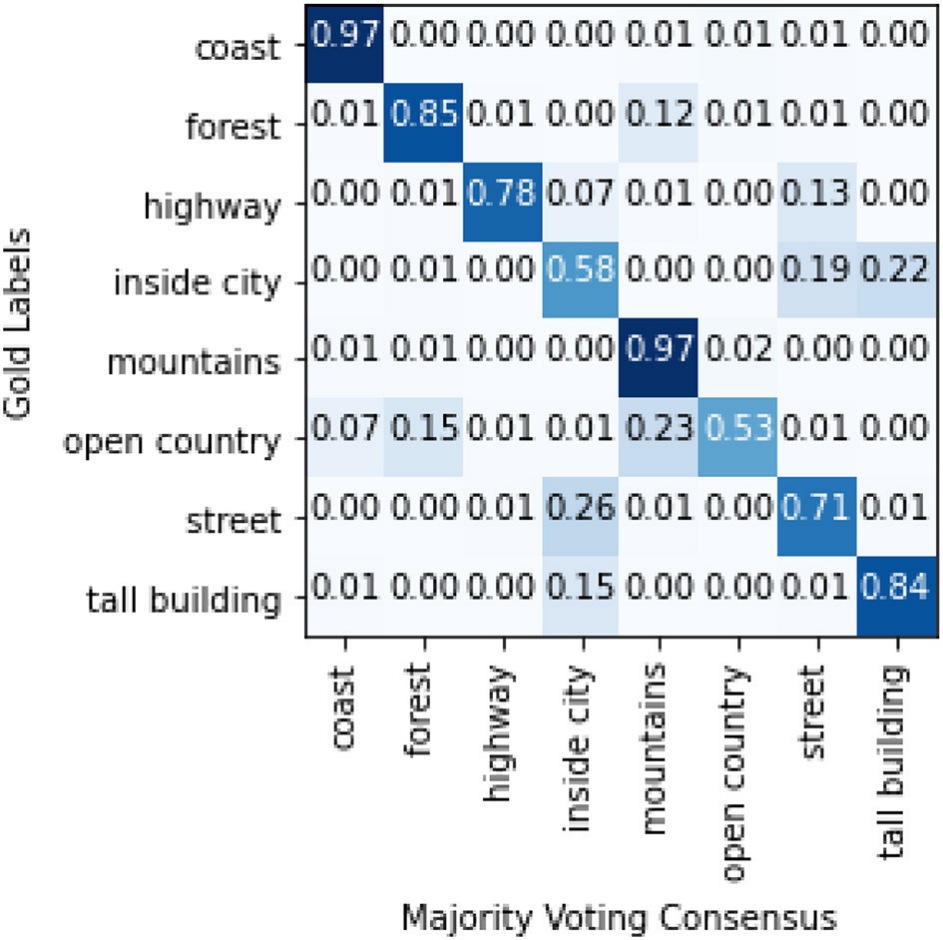
Confusion matrix between gold labels and majority voting consensus for LabelMe.

An alternative explanation can be found in the experiments conducted by Malinin and Gales (2019), who posit that overlapping labels (due to imprecise annotation schemes) introduce **data uncertainty**, resulting in multi-modal distributions^16^. The key characteristic of data uncertainty disagreement is that it is fully observable given the inputs and targets, without the need to appeal to linguistic theory (as in linguistic ambiguity) or annotator background (as in subjectivity disagreement). As such, a network of weights and biases (a machine annotator if you will), given the inputs and label distribution would also experience uncertainty predicting the targets for such images as human annotators do. In fact, an examination of the model's output distribution for the instances with the lowest temperature predictions shows that the model assigned the lowest temperatures (= highest uncertainty) to images belonging to the categories *tall building, street*, or *inside city*, the categories for which the annotators most disagree with the gold (Figure 4 shows the class proportions of images 1st quartile range of temperature while Figure 5 shows the confusion matrix between the majority and the gold). By calibrating its predictions by its level of certainty for each item, the model was able to fine-tune and improve its performance. Again, more research with other datasets characterized by data uncertainty will be required.

## 6. Conclusion and Future Work

Not all disagreements are the same, and it has been shown that not all approaches for learning from disagreement work equally well with datasets containing different types of disagreement (Uma et al., 2021b). In this paper, we reported on experiments on the use of automatic temperature scaling in a learning-from-disagreements setting as a way for automatically adjusting a model to take into account the peculiarities of a particular dataset. Our results show that model calibration *via* automatic temperature scaling can be a simple yet effective approach to improving model performance, particularly with learning ground truth predictions, but only with high disagreement datasets where the disagreements are due to overlapping labels.

We analyzed the temperature values of the successful model in a dataset of this type, to find that the temperature values have some correlation with two known measures of item disagreement/uncertainty—a positive correlation of about 0.3 with an observed agreement and a negative correlation of about 0.3 with entropy. We also observed that the model assigns the lowest temperature to instances with one of the three categories *inside city, street, tall building* shown by Uma et al. to be overlapping. We also found, however, that in datasets where disagreement is due to different reasons, the approach does not work so well.

We provide two possible explanations: automatic temperature scaling provides a good model of uncertainty when disagreements are due to random noise, but not when they are due to biases and automatic temperature scaling is a good indicator of data uncertainty. Further research is however needed to test these explanations with other datasets with the same characteristics.

## Data Availability Statement

Publicly available datasets were analyzed in this study. This data can be found at: https://zenodo.org/record/5130737#.YP_V9o5KiUk.

## Author Contributions

AU: conceptualization, methodology, software, formal analysis, investigation, visualization, and writing. DA: software, investigation, data curation, and writing. MP: conceptualization, methodology, writing—review and editing, supervision, project administration, and funding acquisition. All authors contributed to the article and approved the submitted version.

## Funding

AU and MP are supported by the DALI project, ERC Advanced Grant 695662 to MP. DA was supported by a studentship from the Saudi government.

## Conflict of Interest

The authors declare that the research was conducted in the absence of any commercial or financial relationships that could be construed as a potential conflict of interest.

## Publisher's Note

All claims expressed in this article are solely those of the authors and do not necessarily represent those of their affiliated organizations, or those of the publisher, the editors and the reviewers. Any product that may be evaluated in this article, or claim that may be made by its manufacturer, is not guaranteed or endorsed by the publisher.

## Footnotes

^1^After some experimentation, and in keeping with the other loss function, we decided to use the sum of the squared errors as opposed to the mean.

^2^Malinin and Gales (2019) use the term **data uncertainty** for this type of noise, but as far as we know their notion of data uncertainty is the same as what Reidsma and Carletta (2008) call random noise.

^3^In reverse kl, the target human-derived soft label and the predicted soft label are swapped.

^4^Sigmoid, ReLu, and Tanh outputs are bounded between [0, 1], [0,1], and [–1, 1] respectively, while Softplus outputs are only lower bounded are zero.

^5^Guo et al. (2017) propose the use of the *max*(·) function, rather than *softplus*(·).

^6^As such, it would be more appropriate to name *T*_*i*_ “confidence” or “certainty”—but we will stick with the original name to acknowledge the intellectual debt of our proposal to temperature scaling.

^7^Ties were broken by making a random selection.

^8^ https://github.com/dali-ambiguity

^9^DO judgments with different antecedents are considered identical, and the judgments other than DN or DO are removed.

^10^Another example of corpus were the disagreement is due to linguistic ambiguity is Dumitrache et al. (2019).

^11^ http://labelme.csail.mit.edu/Release3.0

^12^ https://github.com/jcpeterson/cifar-10h

^13^This model was developed for fine-grained information status classification on the isnotes corpus (Markert et al., 2012; Hou et al., 2013).

^14^ https://github.com/KellerJordan/ResNet-PyTorch-CIFAR10

^15^Significance was computed using bootstrap sampling, following Berg-Kirkpatrick et al. (2012) and Søgaard et al. (2014).

^16^The results from Table 1 while not significant do suggest that of the datasets examined in this work, labelme has the most data uncertainty.
